# Retooling CalEnviroScreen: Cumulative Pollution Burden and Race-Based Environmental Health Vulnerabilities in California

**DOI:** 10.3390/ijerph15040762

**Published:** 2018-04-16

**Authors:** Raoul S. Liévanos

**Affiliations:** Department of Sociology, University of Oregon, Eugene, OR 97403-1291, USA; raoull@uoregon.edu

**Keywords:** cumulative impact, ambient air pollution, environmental health, environmental justice, race, Latina/o, segregation, spatial analysis, CalEnviroScreen, California

## Abstract

The California Community Environmental Health Screening Tool (CalEnviroScreen) advances research and policy pertaining to environmental health vulnerability. However, CalEnviroScreen departs from its historical foundations and comparable screening tools by no longer considering racial status as an indicator of environmental health vulnerability and predictor of cumulative pollution burden. This study used conceptual frameworks and analytical techniques from environmental health and inequality literature to address the limitations of CalEnviroScreen, especially its inattention to race-based environmental health vulnerabilities. It developed an adjusted measure of cumulative pollution burden from the CalEnviroScreen 2.0 data that facilitates multivariate analyses of the effect of neighborhood racial composition on cumulative pollution burden, net of other indicators of population vulnerability, traffic density, industrial zoning, and local and regional clustering of pollution burden. Principal component analyses produced three new measures of population vulnerability, including Latina/o cumulative disadvantage that represents the spatial concentration of Latinas/os, economic disadvantage, limited English-speaking ability, and health vulnerability. Spatial error regression analyses demonstrated that concentrations of Latinas/os, followed by Latina/o cumulative disadvantage, are the strongest demographic determinants of adjusted cumulative pollution burden. Findings have implications for research and policy pertaining to cumulative impacts and race-based environmental health vulnerabilities within and beyond California.

## 1. Introduction

### 1.1. Background

Episodic political and legal disputes occurred in the 1960s and 1970s in the United States over environmentally hazardous working and residential conditions for various nonwhite and low-income populations [1,2]. The environmental justice (EJ) movement emerged in the 1980s over the siting of hazardous land uses in socially marginalized communities, and it motivated a nationwide study that argued a community’s nonwhite composition was a primary indictor of hazardous waste location [3]. Prominent EJ and civil rights advocate, Reverend Benjamin Chavis, associated these findings in 1992 with the broader problem of “environmental racism.” This problem is typically understood as the unequal enforcement of environmental laws; systematic exclusion from environmental science, movements, and policy making; and the disproportionate burden of environmental health threats experienced by racial minorities where they live, work, play, pray, and learn [4,5,6]. The Clinton Administration passed federal EJ legislation to address claims of the EJ movement in the 1990s, and many states followed suit despite significant opposition from public administrators and business representatives [7,8].

EJ claims also resonated with audiences across several scholarly realms. For example, historians and social, environmental, and health scientists linked the problem of environmental racism, and its associated environmental health disparities, to the enduring spatial relationships between environmental hazards and segregated nonwhite residential settlements in the United States [6,9,10,11,12,13,14,15,16]. Environmental and public health researchers spanning academia, national scientific entities, and various regulatory agencies also critiqued traditional environmental health risk assessment frameworks and policies that focus too narrowly on single chemical exposures and ignore the social determinants of environmental health [13,17,18,19,20,21,22]. Instead, they advanced the study of “cumulative impacts”: the multiplicity of chemical exposures and effects that people and places experience, and the relationship between those experiences and pre-existing biological, physiological, and social conditions of a human settlement [23].

California is a nationally and internationally prominent site for advancing novel cumulative impact analyses, which are linked to the state’s precedent-setting EJ and climate policies [5,18,24,25,26,27,28,29,30,31,32,33]. Legislation passed in 1999 created the legal definition of EJ in California as “the fair treatment of people of all races, cultures, and incomes with respect to the development, adoption, implementation, and enforcement of environmental laws and policies” [27] (p. 489). The following working definition of cumulative impact emerged in the California Environmental Protection Agency’s (Cal/EPA) multi-stakeholder EJ policy process during the 2000s and reflects established notions of cumulative impact in the scientific literature wherein environmental health disparities are linked to biological, physiological, social, and environmental factors:

“Cumulative impacts means exposures, public health or environmental effects from the combined emissions and discharges in a geographic area, including environmental pollution from all sources, whether single or multi-media, routinely, accidentally, or otherwise released. Impacts will take into account sensitive populations and socio-economic factors, where applicable and to the extent data are available” [34] (p. 648).

This definition of cumulative impact developed alongside the emergence of innovative cumulative impact mapping techniques in California that preceded the EJ screening tool released by the U.S. Environmental Protection Agency (U.S. EPA, Washington, DC, USA) in 2015 [35]. Four prominent cumulative impact mapping tools in California are the Cumulative Environmental Hazard Inequality Index (CEHII) [36,37], the Environmental Justice Screening Method (EJSM) [35,38,39] and its associated Climate Change Vulnerability Screening Method (CCVSM) [25], the Cumulative Environmental Vulnerability Assessment (CEVA) [26,40], and the California Community Environmental Health Screening Tool (CalEnviroScreen) [34,41,42,43]. These cumulative impact mapping techniques similarly model the spatial concentration of chemical and nonchemical environmental stressors, as well as “intrinsic” genetic susceptibilities and health conditions and “extrinsic” social vulnerability factors that are assumed to exacerbate the effects of environmental stressors [23].

The CEHII, EJSM, CEVA and CalEnviroScreen have also informed scholarly and public deliberation over how best to identify heavily impacted communities and the extent to which financial and other resources should be channeled into those communities to improve their health and wellbeing. CalEnviroScreen is noteworthy in this regard. Researchers used CalEnviroScreen to model ovarian cancer survival rates [44], predict spatial patterns in disease burden [45], evaluate electrical grid siting alternatives [46], and corroborate environmental racism and cumulative impact claims by community members opposing the siting of a new hazardous waste facility in their community [47]. In addition, CalEnviroScreen is the only screening tool among these four with statewide coverage, and it is meant to assist the State of California in locating the most environmentally burdened, “disadvantaged communities,” for the targeted receipt of a quarter of the Greenhouse Gas Reduction Funds obtained through California’s carbon cap-and-trade program [33]. Thus, CalEnviroScreen is unique in its statewide coverage, scholarly relevance, and political and economic implications.

### 1.2. Objectives and Hypotheses

**Figure 1:**
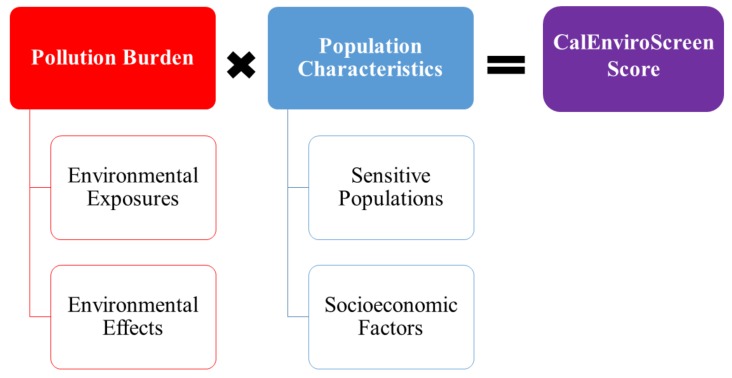
Multiplicative approach of CalEnviroScreen 2.0 (original adaptation from Faust et al. [43]).

CalEnviroScreen is distinct in other ways that are of central concern to the present study. CalEnviroScreen, version 1.0, released at the zip code level in 2013, built upon earlier pilot studies [17,18,28,30,34,41] and modeled cumulative impacts using the multiplicative framework displayed in Figure 1. CalEnviroScreen 1.0 followed established environmental inequality and public health literature, and it paralleled the CEHII, EJSM and CEVA tools by including neighborhood racial composition—i.e., the percent of nonwhite and Latina/o residents—as one of its extrinsic socioeconomic indicators. Other socioeconomic indicators included the spatial concentration of limited educational attainment, limited English-speaking ability, and relative poverty from the American Community Survey. These indicators were combined into a composite cumulative impact score by standardizing them with sensitive population indicators (the spatial concentration of the young and elderly, asthma-induced emergency room visits, and low infant birth weight) and pollution burden indicators measuring environmental exposures and environmental effects (air-, water-, and land-based emissions and contamination). 

The first matter of concern to the present study is that subsequent versions of CalEnviroScreen diverged from the scholarly literature and comparable California cumulative impact screening methods by excluding its racial composition variable. There remains some speculation over the removal of the race variable. This is especially true for EJ advocates who maintain that racial factors significantly and independently influence the distribution of cumulative environmental health burdens in California. EJ advocates’ claims are supported by environmental health and sociological research, which generally finds that nonwhite racial segregation is a “structural factor” in spatially concentrating socioeconomic disadvantage, poor health outcomes, and disproportionate environment pollution burden [11]. These race-based environmental health vulnerabilities are often attributed to the legacy of systematic housing market discrimination, real estate steering practices, and blocked channels of residential and economic mobility, which tend to place nonwhites, especially blacks and Latinas/os, at heightened risk of exposure to industrial and transportation-related health hazards in California and through much of the United States [1,2,3,4,5,6,9,10,11,12,13,14,15,16].

The Office of Environmental Health Hazard Assessment (OEHHA) is Cal/EPA’s primary scientific organization and lead agency on developing CalEnviroScreen [17,18,34,41]. Its stated mission “is to protect and enhance public health and the environment by scientific evaluation of risks posed by hazardous substances” [48]. OEHHA removed race from CalEnviroScreen 1.1 to make it more widely applicable to state entities prohibited from including racial factors in their decision-making processes [49]. However, OEHHA committed to conducting supplemental analyses on the correlation between racial composition, pollution burden, and composite cumulative impact scores in version 1.1 and subsequent versions of CalEnviroScreen [49].

Cushing et al. [42] analyzed data from CalEnviroScreen 1.1 and 2010 decennial census data on racial composition at the zip code level. That study found that the percent of nonwhite populations, especially those identifying as Latina/o or black, was positively associated with cumulative impact scores. The magnitude of the disparities vis-à-vis the state’s non-Latina/o white population uncovered in that study indicated that Latinas/os, followed by blacks, are most likely to live in zip codes that had the highest level of susceptible populations, social vulnerability, and total pollution burden. These relationships were robust when controlling for population density and comparing outcomes between additive and multiplicative cumulative impact scoring procedures. 

OEHHA’s [50] supplemental analysis of census tract-level data in CalEnviroScreen 2.0 found similar disparities for neighborhoods with higher concentrations of Latinas/os and blacks as found in CalEnviroScreen 1.1. However, no study to date has extended such inquiry with multivariate regression analyses of CalEnviroScreen 2.0 despite its developers’ call to pursue such analysis [34,41,42]. Multivariate analyses have been completed on the recently released CalEnviroScreen 3.0, but that study did not include local racial composition in the analyses [45].

This article seeks to address these research gaps and “retool” CalEnviroScreen 2.0 (the most current version of the screening tool at the time this analysis was completed) using publicly available data sources that permit full replication of the analysis presented below. This article assesses the bivariate relationships between individual pollution burden indicators and the cumulative pollution burden score. It applies conceptual frameworks from environmental health and inequality literature and develops an adjusted measure of cumulative pollution burden from the CalEnviroScreen 2.0 data that excludes traffic density and facilitates multivariate analyses of the effect of neighborhood racial composition on cumulative pollution burden, net of other predictors of concentrated environmental health hazard. Principal component analyses are employed to reduce correlated population vulnerability indicators into the three separate composite measures at the census tract level. Economic disadvantage signifies the correlations between limited educational attainment, relative poverty, and unemployment. Black cumulative disadvantage represents the consistent associations between the concentration of blacks, economic disadvantage, low birth weight, and asthma-related hospitalizations. Latina/o cumulative disadvantage similarly reflects the consistent relationship between the concentration of Latinas/os, economic disadvantage, limited English-speaking ability, low birth weight, and asthma-related hospitalizations.

The final aspect of the analysis uses spatial error regression models to assess the effects of the population vulnerability indicators on percentiles of the adjusted cumulative pollution burden indicator, net of other predictors of environmental health hazard concentration. Those other predictors include regional controls for tract location in select air basins of California and plausible emission sources in CalEnviroScreen (i.e., traffic density) and previously not included in CalEnviroScreen (i.e., the extent of industrial land zoning). The regression analyses test the following four hypotheses derived from previous research [10,11,25,26,36,37,39,40,42,51,52,53,54]. These hypotheses emphasize the significant positive effects that the residential segregation (i.e., elevated tract-level concentrations) of blacks and Latinas/os have independently—and in combination with other intrinsic and extrinsic vulnerability factors—on the concentration of cumulative environmental pollution burden in California and throughout the United States.

- **Hypothesis 1 (H1):** The *nonwhite environmental inequality hypothesis* states that the spatial concentration of blacks and Latinas/os will be positively associated with cumulative pollution burden, net of other population vulnerability, emission, and spatial factors.
- **Hypothesis 2 (H2):** The *black environmental inequality hypothesis* states that the spatial concentration of blacks will be the strongest racial predictor of cumulative pollution burden, net of other population vulnerability, emission, and spatial factors.
- **Hypothesis 3 (H3):** The *Latina/o environmental inequality hypothesis* states that the spatial concentration of Latinas/os will be the strongest racial predictor of cumulative pollution burden, net of other population vulnerability, emission, and spatial factors.
- **Hypothesis 4 (H4):** The *Latina/o cumulative disadvantage hypothesis* states that the spatial concentration of Latinas/os, limited English-speaking ability, economic disadvantage, and health vulnerability will predict more variation in cumulative pollution burden than the spatial concentration of blacks, economic disadvantage, and health vulnerability, net of emission and spatial factors.

This study finds that all measures of population vulnerability used in the regression analyses have positive bivariate associations with adjusted cumulative pollution burden. However, the regression analyses ultimately support the Latina/o environmental inequality hypothesis (H3) and the Latina/o cumulative disadvantage hypothesis (H4). Regression analyses suggest that tract-level concentrations of Latinas/os and Latina/o cumulative disadvantage are the strongest demographic determinants of adjusted cumulative pollution burden, net of other indicators of population vulnerability, traffic density, industrial zoning, and local and regional clustering of cumulative pollution burden. These findings have significant implications for research and policy pertaining to cumulative impacts and race-based environmental health vulnerabilities within and beyond California.

## 2. Materials and Methods

### 2.1. Units of Analysis

Census tracts defined for the year 2010 are the units of analysis used in this article. These units offer less precision in characterizing human residential settlement patterns and environmental health vulnerability than census block groups or census blocks. However, CalEnviroScreen 2.0, released in October 2014, used census tracts in response to public comments that critiqued previous iterations’ use of zip codes [34,41,42,49]. OEHHA [55] justified the use of census tracts based on their finer spatial resolution and concordance with county boundaries, as well as the greater degree of homogenous populations and more reliable demographic data available from the U.S. Census and the American Community Survey at that level of analysis. Additional data sources used in the present study were standardized to 2010 tract definitions to facilitate their merger with the CalEnviroScreen 2.0 data. There were 8035 tracts in the complete CalEnviroScreen dataset, with a mean, minimum, and maximum area of 50.87, 0.0002, and 421.51 km^2^, respectively.

### 2.2. CalEnviroScreen 2.0 Indicators

A detailed description of the data sources, analytical techniques for processing those data, and scientific rationale for each individual environmental exposure, environmental effects, sensitive population, and socioeconomic indicator in CalEnviroScreen 2.0 are discussed elsewhere [43]. In summary, the indicators were chosen because they were current, complete, and valid. They also related to various aspects of Cal/EPA’s cumulative impact definition, were of policy-significance to Cal/EPA, adequately represented pollution burden and population characteristics of California (as understood by CalEnviroScreen developers), and could be estimated reliably at the tract-level.

#### 2.2.1. Environmental Exposures

CalEnviroScreen includes four measures of ambient air pollution exposure. The first two measures derive from the California Air Resources Board (CARB) air monitoring network. High ozone concentration was measured as the three-year average estimate of daily maximum 8-h ozone concentration from 2009 to 2011 that exceeded the 8-h standard of 0.070 parts per million for California [43]. The annual mean concentration of PM_2.5_ from 2009 to 2011 is another CalEnviroScreen indicator of ambient air pollution exposure. CalEnviroScreen also features diesel PM emission estimates from CARB and the San Diego Association of Governments (SANDAG) for on-road and non-road sources for a summer (July) 2010 day measured in kilograms per day. Toxicity-weighted manufacturing facility and off-site incinerator air-toxic concentrations in 2010 represent the fourth indicator of ambient air pollution exposure in CalEnviroScreen 2.0. This indicator came from the Geographic Microdata for all 2010 Toxic Release Inventory (TRI) air releases in the U.S. EPA Risk Screening Environmental Indicators (RSEI) model.

Two additional environmental exposure indicators found in CalEnviroScreen 2.0 are associated with ambient air pollution. The first of which is agricultural pesticide use density, derived from the California Department of Pesticide Regulation pesticide use reporting database. This measure covers the total pounds per tract area of 69 pesticides applied in production agriculture from 2009 to 2011 that are deemed especially hazardous and highly volatile to airborne dispersion. Traffic density, derived from the California Department of Public Health (CDPH) and SANDAG, is another CalEnviroScreen environmental exposure indicator associated with ambient air pollution. It is the summed traffic volume within 150 m of a census tract boundary in 2004, adjusted by road segment length (vehicle-kilometers per hour) and divided by the total road length (kilometers).

The drinking water contamination index was the final and most recent environmental exposure indicator added to CalEnviroscreen 2.0. It is compiled from data managed by the CDPH, the California State Water Resources Control Board (SWRCB), the U.S. EPA, and the U.S. Geological Survey. In summary, the index is calculated by summing percentiles of the average water contaminant concentrations or violation index (i.e., the sum of violations of the Maximum Contamination Level for any chemical contaminant and Total Coliform rule) from one compliance cycle of 2005 to 2013 for drinking water systems in California.

#### 2.2.2. Environmental Effects

CalEnviroScreen 2.0 featured two environmental effects indicators that operationalized water- and land-based contamination in California. Proximate impaired water body pollutants were one such measure. It was calculated from SWRCB data by summing percentiles of pollutants across all water bodies listed as impaired under Section 303(d) of the Clean Water Act as of 2010. All scores were summed at the census tract level so as not to double-count the same pollutant across multiple waterways proximate to the tract. The indicator of proximity-weighted leaking underground storage tank sites represents the second indicator of contamination in the water environment. Derived from SWRCB data, it accounts for all sites with valid geographic coordinates that present an environmental hazard or risk to groundwater.

Three land-based environmental effects indicators were proximate cleanup sites, solid waste sites and facilities, and permitted hazardous waste sites and large quantity generators. Proximate weighted cleanup sites were derived from California Department of Toxic Substances (DTSC) and U.S. EPA data. This indicator includes all sites with valid geographic coordinates, presence of hazardous waste, or possessing potential environmental risk. This measure includes U.S. EPA-defined Superfund sites and California State response sites along with lower-risk cleanup sites in California. Proximate weighted solid waste sites and facilities were derived from California’s Solid Waste Information System; the Closed, Illegal, and Abandoned Disposal Sites Program; and the Department of Resources Recycling and Recovery. This measure includes all confirmed closed, illegal, and abandoned solid waste sites, and active solid waste sites with valid geographic coordinates as of 2010. All permitted hazardous waste sites and large quantity generators with valid geographic coordinates were found in DTSC records. This measure includes high-risk hazardous waste landfills whose permit under the Resource Conservation and Recovery Act (RCRA) was expired for at least 10 years or was in interim status. Large quantity generators (i.e., those that produced more than 13.1 tons (i.e., 1000 km) of waste per month for at least one year between 2010 and 2012) and generators producing waste regulated under RCRA were included in the analysis and were weighted based on the annual rate of waste generation.

#### 2.2.3. Cumulative Pollution Burden

As illustrated in Figure 1, the cumulative impact score of CalEnviroScreen 2.0 is the product of the cumulative pollution burden and population characteristic scores—both of which range from 0 to 10. The calculation of the original percentile ranking of tract-level cumulative pollution burden scores in CalEnviroScreen 2.0 are described in Faust et al. [43] and can be summarized as follows. First, the raw values for each environmental exposure and effects indicator were assigned a percentile rank based on their relative value to other values for that indicator for all census tracts in California. The environmental effects indicators received half weights (i.e., multiplied by 0.50) because they were believed to contribute less to the cumulative pollution burden than the exposure indicators. Census tracts that had no value for an indicator were excluded from the percentile ranking and assigned a 0 percentile score to mitigate against underestimating the relative pollution burden of tracts that were impacted by only select types of environmental exposures and/or effects.

The sum of the seven environmental exposure indicator percentiles and the half-weighted five environmental effect indicator percentiles were then divided by 9.5. That denominator was the sum of the total environmental exposure indicators (7) and the product of half-weighting the total environmental effects indicators (i.e., 0.5 × 5 = 2.5). Tracts had their denominator reduced according to the number of environmental exposure indicators that had missing values (PM_2.5_ concentrations, high ozone concentrations, the drinking water contamination index, and/or facility and incinerator air-toxic hazard levels). The averaged cumulative pollution burden percentiles were scaled to a range of zero to ten by: (1) dividing them by the highest average cumulative pollution burden percentile in California (i.e., 82.49); and (2) multiplying the result by ten. The CalEnviroScreen 2.0 cumulative impact score results from multiplying that product by the similarly-derived population characteristics score. However, cumulative pollution burden scores can be understood on their own terms by using the cumulative pollution score or the percentile ranking of that score based on its relative value to other cumulative pollution scores for all 8035 California census tracts that were included in the analysis.

The CalEnviroScreen 2.0 cumulative pollution burden measure helps to identify where multiple environmental exposures and effects concentrate in physical space. However, including traffic density in the cumulative pollution burden measure may be problematic because traffic density is not a pollutant, per se. To be sure, exposure to dense traffic is associated with adverse human health outcomes [43,56], and traffic density can be a valid proxy for near-road air pollution, particularly in the context of air monitoring networks that are ill-suited to track such forms of air pollution [57,58,59].

However, California’s air monitoring network is rather dense [43]. In addition, similar to what is found elsewhere, traffic density is an established *source* of PM_2.5_ and diesel PM [60] and ozone-forming precursors [61], and thus the ambient air pollution that contributes significantly to cumulative pollution burden in California as shown below. Including traffic density in the cumulative pollution burden measure thus limits one’s ability to empirically assess the extent to which traffic density is a predictor of cumulative pollution burden, net of other factors, in California. This study therefore uses an adjusted cumulative pollution burden percentile ranking that excludes the traffic density. Accordingly, *six* environmental exposure indicator percentiles were summed with the half-weighted five environmental effect indicator percentiles and were divided by 8.5, except for when the denominator was reduced due to missing values for a given environmental exposure indicator. The average adjusted cumulative pollution burden percentile was then scaled by dividing it by the highest average adjusted cumulative pollution burden score (i.e., 82.95) and multiplying the result by ten. The adjusted cumulative pollution burden score was assigned a percentile rank based on its relative value to other cumulative pollution scores for all 8035 California census tracts. The remainder of this study used the adjusted cumulative pollution burden percentile ranking as its main dependent variable.

**Figure 2:**
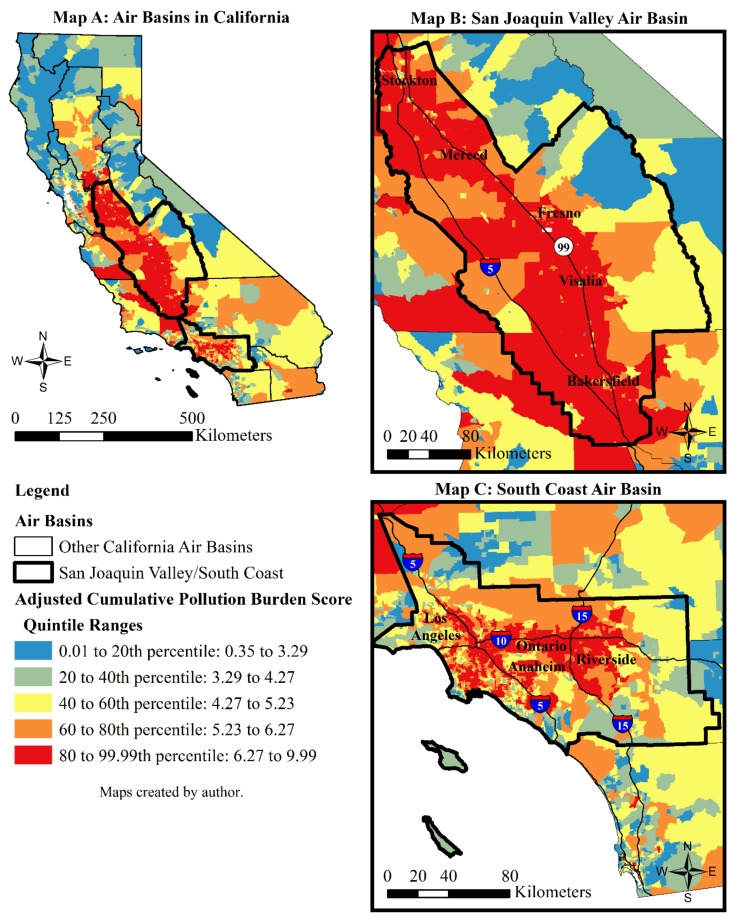
Quintile ranges of adjusted cumulative pollution burden without traffic density in California, the South Coast air basin, and the San Joaquin Valley air basin.

Figure 2 shows the quintile ranges of the adjusted cumulative pollution burden rankings for 8035 census tracts in California. A visual inspection of the maps suggests that elevated levels of adjusted cumulative pollution burden are concentrated in the San Joaquin Valley (SJV) and South Coast air basins. Additional bivariate correlations support this initial observation. Using data for the 15 air basins of California [62] that are discussed further below, it was found that the percentile rankings of adjusted cumulative pollution burden are only positively correlated with the percent of tract intersection in the South Coast air basin (Pearson r = 0.525, *p* < 0.001) and the percent of tract intersection with the SJV air basin (Pearson r = 0.284, *p* < 0.001) (*N* = 8035).

#### 2.2.4. Sensitive Populations

CalEnviroScreen’s indicators of sensitive populations represent the intrinsic biological and physiological susceptibilities and pre-existing health conditions that are believed to amplify the effects of environmental stressors [23]. Children and elderly populations are sensitive receptors for environmental stressors, and they are represented with 2010 Decennial Census data on the percent of population in a tract that is less than 10 or over 65 years of age [43]. Health vulnerability is another indicator of sensitive populations, and it was operationalized in two ways from CDPH data [43]. In one instance, it was a spatially modeled, average age-adjusted rate of emergency department visits for asthma per 10,000 people from 2007 to 2009. In another instance, health vulnerability was measured with a spatially-referenced indicator of low birth weight (LBW) births. This measure was derived by using California birth records on the extent of live, singleton births from 2006 to 2009 that weighed less than 2500 grams. Mothers’ residential addresses at birth were used to geocode the births.

#### 2.2.5. Socioeconomic Factors

The extrinsic, socioeconomic factors in CalEnviroScreen 2.0 were all derived from five-year average estimates from 2008 to 2012 in the American Community Survey. They are all reflected as estimated percentages of the population or households in a tract. Estimates were considered reliable and incorporated into CalEnviroScreen if their standard error was less than half the estimate or were less than the mean standard error for all indicator estimates for California census tracts. The socioeconomic indictor, linguistic isolation, is measured in CalEnviroScreen as the percent of households in tract in which no individual of at least 14 years of age speaks English “very well” or speaks English only. It is highly correlated with the presence of nonwhite populations in a census tract and is a predictor of elevated pollution burden, especially ambient air pollution burden, throughout the United States [52].

The remaining three socioeconomic indicators represent various aspects of economic disadvantage and vulnerability of neighborhood exposure to environmental health hazards [36,37,39,40,52]. The first of which is limited education attainment, measured in CalEnviroScreen as the percent of tract population over 25 years of age with less than a high school education. Second, the tract unemployment rate is represented by the percent of tract population over 16 years that are unemployed and eligible for the labor force. Lastly, the tract relative poverty rate is operationalized as the percent of tract population whose income in the previous year is below two times the federal poverty level (e.g., a single household income of $21,000 a year), which is thought to be an important threshold for households to secure basic necessities [36].

### 2.3. Data External to CalEnviroScreen 2.0

Additional publicly-available data sources external to CalEnviroScreen 2.0 were used to analyze the relationship between adjusted cumulative pollution burden and other tract-level measures not included in the screening tool but that are established correlates of concentrated environmental health hazard within and beyond California. These additional data sources are discussed in more detail below.

#### 2.3.1. Racial Composition

Previous research on CalEnviroScreen 1.1 at the zip code level [42] and CalEnviroScreen 2.0 at the tract level [50] indicate that cumulative impact scores had stronger positive associations with the concentration of Latinas/os and blacks than any other racial group. Racial composition measures were derived from 2010 Decennial Census data to establish the primary tract-level racial statuses that are associated with cumulative pollution burden in California. Consistent with previous research on spatial patterns of racial residential segregation and environmental inequality outcomes using the census data [63,64], this portion of the analysis used the percent of tract population that identifies as Latina/o and separate measures of the tract population that identify as non-Latina/o and white, black, American Indian, Asian, Pacific Islander, some other racial classification, or multiracial. Bivariate analyses that used these measures of racial composition indicated that the percentile rankings of both the CalEnviroScreen 2.0 and the adjusted cumulative pollution burden scores were only positively correlated with the percent of tract population that identified as Latina/o and the percent of tract population that identified as non-Latina/o black. Accordingly, the remainder of the analyses used these two racial composition indicators of population vulnerability. Doing so facilitated tests of the four hypotheses described in Section 1.2.

#### 2.3.2. Industrial Zoning

This study also supplements CalEnviroScreen 2.0 data with a tract-level measure of industrial zoning designations as of 2004 from the first ever statewide general plan database for the State of California created by the California Resources Agency and the University of California, Davis [65]. Polygons coded as “industrial, which includes heavy industry and light industry,” were extracted from the 2004 general plans shapefile and intersected with 2010 census tract boundaries to derive a measure of the percent of tract area that was zoned industrial. There are two main benefits of including this measure in the analysis. First, many CalEnviroScreen pollution burden indicators reflect the adverse environmental health hazards of industrial activity, but none of them operationalize the extent to which census tracts are *zoned* industrial. California land use guidance and social science and historical research indicates that industrial zoning is an important determinant of where environmental health hazards are found [5,11,16,66]. Second, this measure temporally coincides with the 2004 traffic density variable described above—both of which are used as complimentary “emission source” predictor variables in the bivariate and multivariate analyses of variation in adjusted cumulative pollution burden percentiles. Assessing the relative effects of those temporally-matched traffic density and industrial zoning indicators have on subsequent pollution burden can further inform regulatory considerations of how best to address cumulative pollution burden in California census tracts.

#### 2.3.3. Regional Controls: Tract Containment in California Air Basins

Additional measures were used to account for the spatial clustering of adjusted cumulative pollution burden in California. The selection of these measures considered several factors, including the regional clustering of adjusted cumulative pollution burden summarized above and previous analyses of zip code-level CalEnviroScreen 1.1 data, which found that the San Joaquin Valley (SJV) and Greater Los Angeles area in Southern California had the highest cumulative impact scores among regions in the state [42]. In addition, complimentary cumulative impact mapping studies using the CEHII [36,37], the CEVA [26,40], and the EJSM [25,35,38,39] focus on both regions given their significant environmental health risks and unequal cumulative pollution burdens for nonwhites and other socially-marginalized populations. Given these insights and previous environmental inequality research on the social and spatial dimensions of cumulative ambient air pollution burdens in California [67], the spatial regression analyses featured below include separate variables for the percent of tract area that intersects boundaries for the SJV and South Coast air basins [62]. These and the 13 other California air basins are defined in state statute, and they are differentiated by their meteorological, geographic, and air pollution and transport characteristics. The SJV air basin includes Fresno, Kern, Kings, Madera, Merced, San Joaquin, Stanislaus and Tulare Counties. The South Coast air basin includes Los Angeles, Orange, Riverside, San Bernardino, and Ventura Counties.

### 2.4. Analytic Strategy

**Figure 3:**
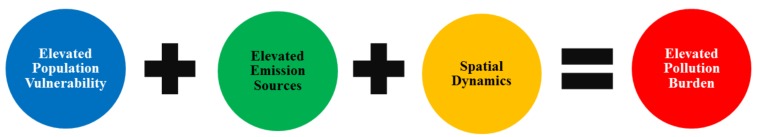
Conceptual framework for modeling the factors that predict adjusted cumulative pollution burden patterns.

This section summarizes the analytic strategy used in the remainder of this study. First, principal component analyses (PCA) specify dimensions of population vulnerability that are significantly associated with adjusted cumulative pollution burden. A series of bivariate correlation analyses demonstrate the validity of the adjusted cumulative pollution burden measure. Then, spatial error regression models assess the effects of population vulnerability factors on adjusted cumulative pollution burden, net of emission sources (i.e., industrial zoning and traffic density) and spatial dynamics (i.e., spatial dependencies in the model parameters and the SJV and South Coast air basin regional controls). Figure 3 visualizes the conceptual framework that guides the spatial regression analyses. 

#### 2.4.1. Principal Component Analyses

Many population vulnerability indicators featured in this study are highly correlated with each other. Principal component analysis (PCA) is one approach used in the social science environmental inequality literature to identify and mitigate such issues of collinearity between population vulnerability indicators [52,53]. Substantively speaking, PCA can also be a useful tool to develop composite measures that represent underlying and complex dimensions of population vulnerability (or other analytical constructs) that cannot be captured using separate indicators of vulnerability [45]. The PCAs included the Latina/o and non-Latina/o black tract composition variables, and all population vulnerability indicators in CalEnviroScreen, except for the children and elderly measure. The incorporation of those variables into the PCA was justified by the common correlation between them and cumulative pollution burden as demonstrated below, as well as established sociological conceptualizations of how various neighborhood-level demographic variables combine to create social disadvantage and vulnerability of exposure to environmental hazards [52,53,68].

In each PCA, unrotated factor solutions were extracted by analyzing the correlation matrix involving each component variable in a maximum of 25 iterations and using eigenvalues greater than 1.00. Missing values were excluded through listwise deletion. A regression method was then used to produce each factor score, which can be interpreted as the standardized linear combination of factor loading weights for each component variable. The validity of the factors were assessed by estimating the total variance explained by each factor component. Cronbach’s alpha scores calculated from additional scale analyses of the factor components informed assessments of the reliability of the factor results. This approach contrasts, for example, with research that used hundreds of input variables and produced multiple factor solutions of “social vulnerability” to environmental hazards throughout the United States [69]. However, this approach accords with social science environmental inequality literature [52,53] and is further justified given the limited number of inputs used and the ease of interpreting the underlying structure of population vulnerability for these California-based population vulnerability factors.

Table 1 summarizes the factor results from the three separate PCAs. The first factor reduces the highly correlated unemployment rate and the low educational attainment and income variables into a composite measure of “economic disadvantage.” This factor is highly reliable and valid given its high Cronbach’s alpha score (0.776), eigenvalue (2.262), and 75.40 percent of variance explained in the factor components. The economic disadvantage factor is used in the analysis below primarily as a means to address issues of multicollinearity between its components parts while including a multidimensional measure of economic disadvantage that is separate from linguistic isolation and racial composition when modeling the effects of population vulnerability, net of other factors, on adjusted cumulative pollution burden.

The other two factors are limited when compared to related measures of “cumulative disadvantage” in the sociological literature, which capture how institutional mechanisms contribute to the accumulation of disadvantage and vulnerability over time for various racial groups [70]. However, each factor of cumulative disadvantage produced from the PCAs in the present study are fairly reliable and valid analytical constructs, and they represent how multiple dimensions of intrinsic and extrinsic environmental health vulnerability tend to accumulate differentially in California neighborhoods with elevated concentrations of Latinas/os and blacks. Latina/o cumulative disadvantage is the most reliable and valid cumulative disadvantage measure. It has a Cronbach’s alpha score of 0.805 and eigenvalue of 3.836. Further, it explains 54.81 percent of variance in the factor components. Latina/o cumulative disadvantage loads higher on Latina/o composition, linguistic isolation, and all aspects of economic disadvantage (factor loadings ≥ 0.635) and lower on the intrinsic sensitive population measures of percent low birth weights and asthma-related hospitalizations. Black disadvantage has comparably low reliability and validity given its lower Cronbach’s alpha (0.712), eigenvalue (2.930), and percent of variance it explains in its component parts (48.84%). Nonetheless, black cumulative disadvantage loads high on all aspects of economic disadvantage and asthma-related hospitalization rates (factor loadings between 0.858 and 0.720), and moderately-high on black composition and percent low-birth weights.

The health vulnerability measures of asthma-related hospitalizations and percent low-birth weights thus had comparatively lower influence on the composite Latina/o and black cumulative disadvantage factors. As shown in Section 3.1, those health vulnerability measures also had comparatively lower correlations with cumulative pollution burden than socioeconomic and racial composition indicators of extrinsic vulnerability. Accordingly, this study followed previous cumulative impact screening studies of the SJV that had similar findings and used the health vulnerability indicators as a “reference layer” [40]—indicative of how health status amplifies socioeconomic and race-based environmental health vulnerabilities of tract exposure to adjusted cumulative pollution burden.

#### 2.4.2. Spatial Regression Models

Preliminary diagnostic tests typically used in the environmental inequality outcomes literature [53,67,71] revealed that the spatial error variant of the ordinary least squares (OLS) linear regression model was the correct specification to account for spatial effects in regression analyses featured in this study. Those analyses test which race-based environmental inequality hypothesis (i.e., H1, H2, H3, and H4) presented in Section 1.2 best explains variation in tract exposure to adjusted cumulative pollution burden. The spatial error model assumes that the explanatory variables alone do not account for spatial autocorrelation in the model and that there is spatial dependence in the regression errors [71]. The spatial error model uses a maximum likelihood method and addresses the potential efficiency problem of having biased coefficient standard errors estimates [72] with the addition of the spatially weighted error term (λ*We*) in the OLS regression equation. Spatial error models are defined in the following manner, which operationalizes the conceptual framework shown in Figure 3:

$$y=\alpha +\underset{k}{\sum }\beta_{k}X_{k}+\lambda We+u$$

where y represents percentiles of adjusted cumulative pollution burden; α is the constant and β is the coefficient for the *k* number of *X* population vulnerability, emission sources, and air basin regional control variables; λ is the spatial autoregressive coefficient; *W* is the spatial weights matrix; *e* is the random error term from the OLS model; and *u* is the spatially independent error term [72,73].

The spatial weights matrix helps to account for spatial dynamics that mediate the relationships between population vulnerability, emission sources, air basin regional controls, and the outcome measure of adjusted cumulative pollution burden. Specifying the correct spatial weights matrix for the spatial error models involved extensive experimentation and consideration of comparable approaches in previous environmental inequality outcomes research [53,67,71]. Ultimately, a row-standardized second-order queen adjacency spatial weights matrix—when used with the regional controls for tract intersection with the South Coast and SJV air basins—successfully accounted for spatial dependence in the spatial error models. The matrix resulted in only seven “island” tracts without a neighbor out of the 7610 tracts included in the regression analyses. There were a maximum of 71 and mean of 19 neighbors across the remaining 99.91 percent (*N* = 7603) tracts with a neighbor.

The spatial error regression models are estimated for 7610 tracts with non-missing data. Table 2 displays the descriptive statistics for all variables used in those models. The table includes the Moran’s *I* values for each variable, which was calculated using the second-order queen adjacency spatial weights matrix. The Moran’s *I* values indicate all the variables exhibit significant spatial clustering and further justify the use of the spatial error regression approach in this article.

## 3. Results

### 3.1. Bivariate Analyses

Table 3 displays the Pearson correlation estimates between the CalEnviroScreen 2.0 and adjusted cumulative pollution burden percentiles and the percentiles of individual pollution indicators used in CalEnviroScreen 2.0. Percentile rankings do not permit one to discern the absolute magnitude of pollution burden differences between census tracts, but they are central scoring techniques in CalEnviroScreen 2.0 for stratifying tracts according to relative pollution burden in California [43].

Table 3 displays substantively similar positive associations between the individual pollution burden indicators and the CalEnviroScreen 2.0 and adjusted cumulative pollution burden indicators. The individual environmental exposures and effects indicators have differing associations with cumulative pollution burden, as found in early CalEnviroScreen pilot data [41]. Both cumulative pollution burden measures had the highest bivariate correlations with annual mean PM_2.5_ concentrations, toxicity-weighted manufacturing facility and off-site incinerator air-toxic concentrations, and diesel PM emissions. These patterns indicate the importance of ambient air pollution in contributing to concentrated cumulative pollution burdens in California. The drinking water contamination index has a moderate correlation with both pollution burden indicators, while high ozone and pesticide application concentrations have lower associations with cumulative pollution burden. In addition, cumulative pollution burden percentile rankings using the CalEnviroScreen 2.0 and the adjusted approach that excludes traffic density are almost identical with a Pearson correlation coefficient of 0.972 (*p* < 0.001).

The biggest change evidenced in Table 3 is that traffic density has a moderate correlation with CalEnviroScreen 2.0 cumulative pollution burden but a low correlation with adjusted cumulative pollution burden. Excluding the traffic density variable has little substantive change on the cumulative pollution burden percentile ranking. It also permits an assessment of the effect of traffic density on cumulative pollution burden, which is heavily influenced by ambient air pollution attributable to traffic-related sources [60].

Table 4 displays the extent to which racial composition and the sensitive population and socioeconomic factors included in CalEnviroScreen 2.0 are significantly associated with the percentile rankings of cumulative pollution burden in California. Raw values of the population vulnerability variables, rather than their percentile rankings, facilitate easier interpretation of how the population composition of tracts is associated with relative cumulative pollution burden. Similar to patterns shown in Table 3, Table 4 indicates that the relationship between population vulnerability and the percentile rankings of both cumulative pollution burden indicators are essentially the same.

Table 4 reveals three other noteworthy patterns. First, all the population vulnerability measures in CalEnviroScreen 2.0 are positively associated with CalEnviroScreen 2.0 cumulative pollution burden and adjusted cumulative pollution burden, except for the extent of children and elderly populations in a census tract. This pattern indicates that, while such sensitive populations are at heightened risk of experiencing adverse health conditions from environmental exposures [23,35], the extent of their presence in a census tract is consistently a *negative* predictor of cumulative pollution burden in California. Indeed, CalEnviroScreen 3.0 excludes the children and elderly indicator due to public comments and internal evaluations by CalEnviroScreen developers that acknowledged this fact [74]. Second, the correlations between the other sensitive population indicators relating to tract-level health vulnerability and cumulative pollution burden are positive but smaller in magnitude than the correlations between most of the socioeconomic indicators of CalEnviroScreen and the racial composition measures. This pattern compliments previous research on cumulative pollution burdens in the SJV in which comparable health status measures were marginally related to cumulative pollution burden in comparison to socioeconomic factors [40]. Third, the extent of Latina/o presence in a tract has the highest bivariate correlation with cumulative pollution burden.

Table 5 displays the bivariate correlations between all of the independent variables and percentiles of adjusted cumulative pollution burden that are used in the spatial error regression analysis. As expected, all of the independent variables are positively correlated with adjusted cumulative pollution burden. These correlations are particularly noteworthy in relation to the four hypotheses guiding this study. The percentages of Latina/o population and non-Latina/o black population are both positively associated with adjusted cumulative pollution burden—indicating that the nonwhite environmental inequality hypothesis (H1) may be supported in the subsequent multivariate analysis. However, the magnitude of the association between these racial composition variables and adjusted cumulative pollution burden suggests that the concentration of Latinas/os and cumulatively disadvantaged Latina/o tracts—rather than comparable black census tracts—may be the stronger predictor of concentrated environmental health hazard levels. Such patterns would not support the black environmental inequality hypothesis (H2), but they would support the Latina/o environmental inequality (H3) and Latina/o cumulative disadvantage (H4) hypotheses.

Table 5 further shows that only the population vulnerability variables share concerning levels of intercorrelation. Specifically, economic disadvantage, percent linguistically isolated households, and percent Latina/o population are all strongly correlated with each other. In addition, both Latina/o and black cumulative disadvantage are strongly correlated with each other and the other population vulnerability measures that comprise those cumulative disadvantage factor variables. Those intercorrelations were considered in organizing the spatial error regression models presented below.

### 3.2. Multivariate Analyses

All models shown in Table 6 and Table 7 include the variables that operationalize emission sources and the regional and local clustering of adjusted cumulative pollution burden. Spatial dependence is not evident in those spatial error models as indicated by their highly significant spatial autoregressive coefficient (Lamda; λ) and the insignificant Moran’s *I* statistics for the regression residuals.

Model 1 in Table 6 establishes the effects of socioeconomic factors from CalEnviroScreen 2.0 data on adjusted cumulative pollution burden, net of emission sources and spatial factors. Including emission sources, spatial factors, and economic disadvantage in Model 1 erases the significant and positive bivariate correlation between the percent of linguistically isolated households and adjusted cumulative pollution burden witnessed in Table 5. Instead, economic disadvantage rises as the more consistent population vulnerability predictor of adjusted cumulative pollution burden in Model 1.

Model 2 in Table 6 adds percent Latina/o and non-Latina/o black population to the variables in Model 1 and tests the nonwhite (H1), black (H2), and Latina/o (H3) environmental inequality hypotheses. The multicollinearity condition index (MCI) increases from Model 1 to Model 2 with the inclusion of racial composition. This result is expected given the bivariate intercorrelation between economic disadvantage, percent linguistically isolated households, and percent Latina/o population shown in Table 5. However, the MCI in Models 1 and 2 are well below the suggested threshold of 30 [73] and thus demonstrate the lack of significant collinearity between the independent variables in each of those models.

Percent Latina/o population is the only population vulnerability indicator to have a significant effect on adjusted cumulative pollution burden in Model 2 in Table 6. This finding is noteworthy, as none of the CalEnviroScreen 2.0 socioeconomic factors in Model 2 are significantly associated with percentiles of adjusted cumulative pollution burden. The unstandardized regression coefficient (b) indicates that a one percentage-point increase in Latina/o composition is associated with a 0.06 percentile increase in adjusted cumulative pollution burden. This positive effect of a tract’s Latina/o composition partially supports the nonwhite environmental inequality hypothesis (H1) but fully supports the Latina/o environmental inequality hypothesis (H3). The effect of a tract’s black composition on adjusted cumulative pollution burden is not significant in Model 2. This finding does not support the nonwhite and black (H2) environmental inequality hypotheses.

The model summarized in Table 7 tests the Latina/o cumulative disadvantage hypothesis (H4). The model is structured to avoid multicollinearity issues that would arise by including all other population vulnerability variables shown in Table 6 in the same model with the two cumulative disadvantage variables in Table 7. Despite the high correlation between Latina/o and black cumulative disadvantage, the MCI for the cumulative disadvantage model is low at 5.664. This model supports the Latina/o cumulative disadvantage hypothesis (H4). A one-point increase in Latina/o cumulative disadvantage is significantly associated with a 1.3 percentile increase in adjusted cumulative pollution burden, net of other factors included in the model. The coefficient for black cumulative disadvantage is signed as expected but not significant. Thus, the significant and positive bivariate association between black cumulative disadvantage and adjusted cumulative pollution burden is washed away by the multivariate results presented in Table 7.

The consistent effects of the emission source and regional control variables on adjusted cumulative pollution burden across the regression models in Table 6 and Table 7 are as expected. Traffic density and the extent of industrial-zoned land as of 2004 have highly significant and positive effects on subsequent levels of cumulative pollution burden in a tract. All of the models indicate that a one thousand-point increase in traffic density and a one percentage-point increase in industrial-zoned land are associated, respectively, with a 1.43–1.45 percentile and a 0.28 percentile increase in adjusted cumulative pollution burden. The air basin regional control variables similarly have highly significant and positive effects in the models and thus adequately capture the spatial clustering of adjusted cumulative pollution burden in the South Coast and SJV air basins.

The relative strength of the independent variables in each model can be assessed with unit-less standardized regression coefficients (B). The higher the standardized coefficient, the stronger the effect of an independent variable on adjusted cumulative pollution burden percentiles. The standardized coefficients across the models show that the strongest determinants of adjusted cumulative pollution burden rankings among all variables considered in this analysis are tract containment in the South Coast and SJV air basins, followed by the extent of industrial-zoned land and traffic density in a tract.

The pseudo *R*^2^ is constant across the models in Table 6 and Table 7 at 0.811, which suggests the models account for about 81 percent of the variance explained in the percentile rankings of adjusted cumulative pollution burdens in California census tracts. While informative, the Akaike information criterion (AIC) and the log likelihood statistics have more efficacy in informing comparisons of model fit to the data. In addition, the AIC and log likelihood statistics aid in assessing the relative strengths of the models in their ability to capture how racial composition combines with other factors to explain variation in cumulative pollution burden outcomes. The smaller AIC values and larger log likelihood values shown for Model 2 in Table 6 suggest it best fits the data [71,73]. These findings indicate that the effect of racial segregation on cumulative pollution burden is perhaps best captured when using the percent Latina/o population in a census tract alongside the other indicators of population vulnerability, emission sources, and spatial dynamics included in Model 2. The standardized coefficients in Model 2 suggest that—on average—tract-level variation in percentile ranking of adjusted cumulative pollution burden in California from 2005 to 2013 was most consistently associated with tract containment in South Coast and SJV air basins, the extent of industrial-zoned land and traffic density in a tract in 2004, and the percent of Latina/o population in a tract in 2010.

## 4. Discussion

The U.S. EJ movement emerged in the 1980s over race and class inequities in the distribution of environmental health hazards, environmental enforcement, and exclusionary environmental movements, science, and policy-making. Struggles to address these environmental inequalities continue to this day alongside important opportunities to advance the movement for environmental health equity. This especially true in California, where prominent EJ activism, precedent-setting EJ policies, and innovative EJ researchers have made the state a home for the development of cumulative impact mapping techniques that advance our understanding of the relationship between population vulnerability and the spatial concentration of multiple environmental health hazards [23,28,35].

CalEnviroScreen emerged from this context as a novel cumulative impact mapping tool with broad implications for environmental and public health research and policy. However, it excludes racial status as an indicator of population vulnerability of exposure to multiple environmental health hazards in an effort to make it more widely applicable throughout California state government [49]. As such, it departs from its historical foundations and comparable cumulative impact mapping tools in California [25,26,35,36,37,38,39,40], as well as from a broad cross-section of scholarly literature that emphases the salience of racial factors, particularly racial segregation, in concentrating socioeconomic, health, and environmental disadvantages in the United States and California [1,2,3,4,5,6,9,10,11,12,13,14,15,16]. In addition, CalEnviroScreen is limited in that its cumulative pollution burden indicator precludes researchers and policy makers from assessing the extent to which traffic patterns and/or industrial zoning—well documented pollution sources [5,16,60,61,66]—contribute to the spatial concentration of multiple environmental health hazards in California.

These limitations are manifest in the only other multivariate analysis of CalEnviroScreen to date by Greenfield, Rajan and McKone [45]. That study analyzed zip code-level associations between the CalEnviroScreen 3.0 cumulative impact score and its component environmental and socioeconomic variables. PCA was used in that analysis to uncover multivariate relationships in CalEnviroScreen while reducing the environmental and socioeconomic variables into composite factors and facilitating a spatial regression analysis of their effects on zip code-level disease burden (i.e., the hospitalization rate for at least one of 14 diagnostic categories that have potential environmental etiology). Similar to the present study, that analysis of CalEnviroScreen 3.0 found that industrial activity and air pollution contribute significantly to cumulative impacts in California, and that the SJV and the South Coast region were hot spots for cumulative impacts in the state. It also found that the socioeconomic factor produced from PCA of the CalEnviroScreen 3.0 data explained more of the variance in disease burden than the environmental factor variables. While insightful, such line of inquiry is dependent primarily on CalEnviroScreen data and the course spatial resolution of zip codes. It is further limited by not assessing the effect of racial segregation on cumulative pollution burdens in California, net of non-racial population vulnerability factors, industrial zoning, traffic density, and the spatial clustering of cumulative pollution burden in the state.

The present study “retools” CalEnviroScreen and begins to address these important limitations by incorporating conceptual frameworks and analytical techniques from the environmental health and inequality literature. Such scholarship illuminates the effect of race-based environmental health vulnerabilities on cumulative pollution burden, net of other factors accounted for—and unaccounted for—in CalEnviroScreen.

The resulting analytical framework advanced in this article is analogous to previous sociological efforts to reformulate cross-national analyses of environmental impacts using the “IPAT” model. The IPAT accounting equation specified environmental impacts (“I”) as the product of population (“P”), affluence (“A,” measured as per capita consumption or production levels), and technological factors (“T”). An alternative stochastic approach used in the “STIRPAT” model [75,76] reformulated IPAT and facilitated multivariate regression analyses, which test sociological theories specifying how population, affluence, and technology contribute to environment impacts. Impacts analyzed in that line of inquiry include, for example, national emissions of CO_2_ [77] and ecological footprints (i.e., the amount of biologically productive land needed to sustainably support each individual in a nation) [78].

Similarly, this study retooled CalEnviroScreen by moving away from the multiplicative framework wherein cumulative impacts are the product of cumulative pollution burden (environmental exposures and effects) and population vulnerability (sensitive populations and socioeconomic factors) (see Figure 1). As shown in Figure 3, the present study modeled the adjusted cumulative pollution burden percentile rankings (that excludes traffic density) as a multivariate function of racial and non-racial population vulnerability factors, traffic density and industrial zoning, and local and regional spatial dynamics. Bivariate correlations between CalEnviroScreen’s and this study’s cumulative pollution burden measures, and the individual pollution burden variables that comprise them, show both cumulative pollution burden indicators are substantively similar and heavily influenced by ambient air pollution levels. The primary difference between the cumulative pollution burden indicators results from excluding traffic density in the adjusted measure: traffic density has a moderate correlation with the CalEnviroScreen 2.0 cumulative pollution burden and a lower correlation with adjusted cumulative pollution burden.

This study demonstrates how CalEnviroScreen can be effectively reformulated to address long-lasting EJ concerns [5] and related scholarship on environmental inequality, environmental health disparities, and cumulative impacts regarding the effects of race-based vulnerabilities on the unequal distribution of environmental health hazards [10,11,25,26,36,37,39,40,42,52,54]. As found in previous cumulative impact mapping studies of California’s SJV [40], the extrinsic vulnerability measures of socioeconomic status in CalEnviroScreen and the additional racial composition measures incorporated in this study were more strongly correlated with both cumulative pollution burden indicators than the intrinsic vulnerability measures of health status and susceptible populations. In addition, the percent of non-Latina/o black and Latina/o population in a tract were positively correlated with both cumulative pollution burden indicators. The extent of Latina/o presence in a tract, however, had the strongest bivariate correlation with cumulative pollution burden indicators. These patterns provided initial evidence of the continuing significance of race-based vulnerabilities in effecting cumulative pollution burden in California—regardless of whether traffic density is included in the cumulative pollution burden indicator.

As found in previous related research [45,52,53], principal component analyses helped to reduce highly correlated variables into three dimensions of population vulnerability that were incorporated into the spatial error regression analyses of adjusted cumulative pollution burden. These principal component factors were economic disadvantage and separate cumulative disadvantage factors for black and Latina/o neighborhoods. Black cumulative disadvantage represented the extent of health vulnerability, economic disadvantage, and black populations in a tract. Latina/o cumulative disadvantage represented the extent of health vulnerability, economic disadvantage, limited English-speaking ability, and Latina/o populations in a tract.

The findings from the spatial error regression analyses have important scholarly and practical implications. First, economic disadvantage was the primary population vulnerability determinant of adjusted cumulative pollution burden rankings in California when racial factors were not considered in the regression analysis. However, when a tract’s black and Latina/o composition were considered alongside CalEnviroScreen 2.0 socioeconomic factors of economic disadvantage and linguistic isolation in the regression analysis, the percent of Latina/o population in a tract was the only population vulnerability indicator to have a significant effect on adjusted cumulative pollution burden. In addition, black and Latina/o cumulative disadvantage both had significant positive bivariate correlations with adjusted cumulative pollution burden. However, Latina/o cumulative disadvantage was the only cumulative disadvantage factor to maintain a statistically significant and positive effect on adjusted cumulative pollution burden percentile ranking in the spatial error regression analysis.

These racial patterns compare and contrast with previous cumulative impact mapping studies using CalEnviroScreen. In particular, zip code-level research on CalEnviroScreen 1.1 found that Latinas/os, followed by blacks, were significantly more likely than whites to live in areas with higher cumulative pollution burden, more susceptible populations, and lower socioeconomic status, net of population density and different cumulative impact scoring procedures [42]. Similar race-based environmental health disparities for Latinas/os and blacks were found in bivariate analyses of CalEnviroScreen 2.0 [50]. Racial factors were not considered in the multivariate analysis of CalEnviroScreen 3.0 by Greenfield, Rajan, and McKone [45]. Thus, the present study offers a novel multivariate framework for integrating and analyzing CalEnviroScreen data and data external to the screening tool to advance scholarly and policy-relevant environmental and public health research on race-based environmental health vulnerabilities and cumulative pollution burden in California.

This article illustrates that Latinas/os in California are, on average, more likely to be exposed to social, health, and environmental burdens than other racial groups. This pattern was found even when controlling for a broader set of factors that generally contribute to environmental health disparities in California and throughout the United States [11,52,67], but have yet to be accounted for in previous cumulative impact mapping studies using CalEnviroScreen. In particular, tract containment in the South Coast and SJV air basins, followed by the extent of industrial-zoned land and traffic density in a tract, were the strongest determinants of adjusted cumulative pollution burden rankings among all variables considered in this analysis. These findings support previous regional cumulative impact mapping studies that suggest living in the South Coast and SJV air basins is a significant and independent place-based vulnerability of exposure to cumulative pollution burdens [25,26,35,36,37,38,39,40,42].

However, previous research merely focused on the vulnerability of nonwhite populations in the South Coast and SJV. This study builds environmental inequality research that is attentive to the distinct vulnerabilities of different racial groups [52,79]. Specifically, it demonstrates the need to differentiate relative cumulative pollution burdens across nonwhite residential settlements to more fully assess the nature and extent of race-based environmental health vulnerabilities in California and its South Coast and SJV regions.

Despite the contributions of this study, future research should address its important limitations. First, data in CalEnviroScreen 2.0 and the additional sources used in this study inform our understanding of racial disparities in cumulative pollution burden in California between 2004 and 2013. Environmental health researchers have begun to analyze more recent CalEnviroScreen 3.0 data, and they claim similar associations evident between CalEnviroScreen 2.0 component indicators are found in CalEnviroScreen 3.0 [45]. Future research should examine the extent to which the conceptual framework and findings from this study apply to CalEnviroScreen 3.0 data and more recent land use and racial composition data.

Second, future research with CalEnviroScreen should follow the lead of studies of environmental health vulnerabilities in Texas [80] and Florida [81], which demonstrate the need for environmental health and inequality researchers to untangle the broad “Latina/o” category. Doing so enables one to more fully understand how Latina/o environmental health vulnerabilities are affected by, for example, country of origin, English-speaking ability, income, and education. Data limitations of American Community Survey, such as the limited reliability of some of the average estimates [43], will preclude researchers from fully pursuing such lines of inquiry in the future. However, there are opportunities available to reliably examine differential and spatialized race-based environmental health vulnerabilities if CalEnviroScreen data were merged with U.S. Decennial Census data on, for example, family structure, home ownership, and specific origin for Latinas/os (and other racial groups).

Third, this cross-sectional study did not attend to the important historical processes and events that concentrate cumulative pollution burdens in California neighborhoods. Future research could use urban sociological and environmental inequality literature as a guide [15,16,82,83,84] and examine how segregationist ideologies, state policies, real estate industry actions, and various dynamics of neighborhood change spatially concentrated industrial land uses, major transportation corridors, cumulative pollution burdens, and vulnerable populations in California.

Fourth, this study did not attend to the fine-grained spatial scales, such as individual-level residential mobility patterns throughout a diverse set of spatial contexts overtime [78,85], which may contribute to the environmental health disparities uncovered in the present study. What are the diverse pathways by which individuals are exposed to multiple environmental health hazards over space and time in California?

Lastly, this study may have limited generalizability to other contexts outside of California. Specifically, traffic density may be an important proxy for near-road air pollution in areas outside of California with less densely developed air monitoring networks. In those contexts, cumulative pollution burden may need to incorporate traffic density as a proxy environmental pollution indicator. In addition, this study emphasized the significance of racial segregation, especially of Latinas/os, as a determinant of concentrated cumulative pollution burden, net of other factors. Latina/o cumulative environmental health disparities evident in California are salient, on average, throughout the United States [52]. However, the extent to which the residential segregation of Latinas/os, or blacks or other racial minorities for that matter, best represents population vulnerability and the social determinants of cumulative pollution burden may vary by region throughout the United States and likely throughout various international contexts. A key insight from this study and other cumulative impact mapping studies of California [25,26,35,36,37,38,39,40], however, is that cumulative pollution burdens tend to be concentrated amongst the most vulnerable and segregated populations in a given locality. Researchers and policy-makers must recognize that tendency and direct their research and policy accordingly or they will miss the key social determinants of cumulative pollution burden in their respective area of study and regulatory authority.

## 5. Conclusions

In sum, this study demonstrated how cumulative impact mapping tools, such as CalEnviroScreen, can be merged with additional data to illuminate the enduring significant effect that race-based vulnerabilities have on the distribution of cumulative environmental health hazards. The findings from this study suggest that racial segregation, in general, and the spatial concentration of Latinas/os, in particular, is significantly associated with the cumulative disadvantages of social, health, and environmental burdens in California. These findings support the Latina/o environmental inequality and Latina/o cumulative disadvantage hypotheses regarding the disproportionately high cumulative pollution burdens that Latina/o tracts—including those with multiple forms of socioeconomic disadvantage and health vulnerability—experience above all other racial groups in California. Furthermore, this study found that the extent of Latinas/os in a tract—rather than *any* of the population vulnerability indicators in CalEnviroScreen—was the strongest population vulnerability determinant of cumulative pollution burdens in California. Researchers and policy makers are not privy to such insights when they produce and disseminate color-blind cumulative impact screening tools, such as CalEnviroScreen, even if the intent is to make those tools more applicable throughout a diverse set of decision-making contexts. In addition, this study demonstrates the importance of measuring the effects that different pollution sources and spatial dynamics have on the distribution of cumulative pollution burden in California, as those factors were the strongest overall determinants of cumulative pollution burden in the analysis. Failure to account for those effects may misdirect efforts to promote environmental health equity away from the hot spots of environmental health vulnerability that are most in need of direct regulatory and investment interventions, such as those available through California’s Greenhouse Gas Reduction Fund. The findings from this study suggest that such hot spots are overrepresented in the South Coast and SJV air basins, as well as in neighborhoods throughout the state most consistently comprised of elevated levels of industrial-zoned lands, traffic volume, and segregated and cumulatively-disadvantaged Latina/o populations.

## Figures and Tables

**Table 1:** Principal component analysis results for economic disadvantage, black cumulative disadvantage, and Latina/o cumulative disadvantage.

Variables	Factor Loadings		
	Economic Disadvantage	Black Cumulative Disadvantage	Latina/o Cumulative Disadvantage
Percent of population > 25 without a high school education	0.895	0.773	0.939
Percent of population living below two times the federal poverty level	0.937	0.858	0.914
Percent of the population over the age of 16 that is unemployed	0.763	0.720	0.635
Percent non-Latina/o black population		0.530	
Percent Latina/o population			0.858
Percent linguistically isolated households			0.748
Percent low birth weight births		0.499	0.360
Age-adjusted asthma-related emergency department visits		0.741	0.539
Alpha	0.776	0.712	0.805
Eigenvalue	2.262	2.930	3.836
Percent of total variance explained	75.397	48.840	54.806
*N*	7780	7778	7610

**Table 2:** Descriptive statistics of variables used in the spatial error regression analyses (*N* = 7610 tracts).

Variable	Mean	SD	Min.	Max.	Moran’s *I* ^1^
Adjusted cumulative pollution burden percentile	50.23	28.77	0.01	99.99	0.768 ***
Economic disadvantage	0.00	1.00	−1.71	3.88	0.595 ***
Percent linguistically isolated households	11.25	10.98	0.00	79.10	0.586 ***
Percent Latina/o population	36.95	26.44	1.19	99.03	0.723 ***
Percent non-Latina/o black population	5.79	9.29	0.00	89.76	0.726 ***
Latina/o cumulative disadvantage	0.00	1.00	−1.66	3.12	0.669 ***
Black cumulative disadvantage	0.00	1.00	−1.79	4.76	0.686 ***
Percent industrial-zoned land	4.76	12.08	0.00	93.73	0.162 ***
Traffic density (1000 s)	1.26	1.22	0.00	43.56	0.281 ***
Percent tract in the South Coast air basin	43.40	49.53	0.00	100.00	0.989 ***
Percent tract in the San Joaquin Valley air basin	9.20	28.88	0.00	100.00	0.963 ***

^1^ A second-order queens adjacency spatial weights matrix and 9999 permutations were used in the Moran’s *I* analyses. *** Pseudo *p* < 0.001 (two-tailed test).

**Table 3:** Pearson correlations between percentiles of CalEnviroScreen (CES) 2.0 cumulative pollution burden and adjusted cumulative pollution burden, and individual pollution indicators.

Individual Pollution Indicators	CES 2.0 Cumulative Pollution Burden	Adjusted Cumulative Pollution Burden	N
Environmental exposures			
High ozone	0.342 ***	0.398 ***	7970
PM_2.5_	0.726 ***	0.737 ***	7944
Diesel PM	0.560 ***	0.487 ***	8035
Facility & incinerator air-toxic hazard	0.604 ***	0.572 ***	8023
Agricultural pesticide density	0.125 ***	0.190 ***	8035
Traffic density	0.396 ***	0.181 ***	8035
Drinking water contamination index	0.505 ***	0.572 ***	8000
Environmental effects			
Impaired water body pollutants	0.026 *	0.023 *	8035
Leaking underground storage tank sites	0.312 ***	0.306 ***	8035
Cleanup sites	0.435 ***	0.438 ***	8035
Solid waste sites & facilities	0.271 ***	0.302 ***	8035
Hazardous waste facilities & large quantity generators	0.468 ***	0.446 ***	8035

* *p* < 0.05; *** *p* < 0.001 (two-tailed test).

**Table 4:** Pearson correlations between percentiles of CalEnviroScreen (CES) 2.0 cumulative pollution burden and adjusted cumulative pollution burden and population vulnerability indicators.

Population Vulnerability Variables	CES 2.0 Cumulative Pollution Burden Percentile	Adjusted Cumulative Pollution Burden Percentile	N
Sensitive populations			
Percent of population <10 and >65 years old	−0.119 ***	−0.105 ***	8024
Percent low birth weight births	0.137 ***	0.133 ***	7994
Age-adjusted asthma-related emergency department visits	0.101 ***	0.117 ***	8035
Socioeconomic factors			
Percent of population > 25 without a high school education	0.381 ***	0.404 ***	7922
Percent of population living below two times the federal poverty level	0.310 ***	0.334 ***	7933
Percent of population over the age of 16 that is unemployed	0.154 ***	0.184 ***	7854
Percent linguistically isolated households	0.347 ***	0.337 ***	7719
Racial composition			
Percent Latina/o population	0.446 ***	0.465 ***	8024
Percent non-Latina/o black population	0.074 ***	0.058 **	8024

** *p* < 0.01; *** *p* < 0.001 (two-tailed test).

**Table 5:** Pearson correlation coefficients for variables used in the spatial error regression analyses (*N* = 7610 tracts).

	1	2	3	4	5	6	7	8	9	10
1. Adjusted cumulative pollution burden percentile										
2. Economic disadvantage	0.36 ***									
3. Percent linguistically isolated households	0.34 ***	0.65 ***								
4. Percent Latina/o population	0.47 ***	0.78 ***	0.63 ***							
5. Percent non-Latina/o black population	0.06 ***	0.21 ***	−0.00	0.04 ***						
6. Latina/o cumulative disadvantage	0.41 ***	0.96 ***	0.75 ***	0.86 ***	0.24 ***					
7. Black cumulative disadvantage	0.31 ***	0.90 ***	0.51 ***	0.66 ***	0.53 ***	0.91 ***				
8. Percent industrial-zoned land	0.31 ***	0.19 ***	0.14 ***	0.22 ***	0.05 ***	0.21 ***	0.18 ***			
9. Traffic density (1000 s)	0.15 ***	−0.01	0.10 ***	0.07 ***	0.06 ***	0.03 **	−0.00	0.11 ***		
10. Percent tract in the South Coast	0.54 ***	0.10 ***	0.24 ***	0.28 ***	0.08 ***	0.16 ***	0.07 ***	0.12 ***	0.17 ***	
11. Percent tract in the San Joaquin Valley	0.29 ***	0.23 ***	0.01	0.15 ***	−0.05 ***	0.19 ***	0.20 ***	0.02	−0.15 ***	−0.28 ***

** *p* < 0.01; *** *p* < 0.001 (two-tailed test).

**Table 6:** Spatial error regression results for adjusted cumulative pollution burden percentile on population vulnerability (socioeconomic factors and racial composition), emission sources, and air basin regional controls (*N* = 7610 tracts).

Variables	Model 1 ^1^			Model 2 ^2^		
	b	S.E.	B	b	S.E.	B
Population vulnerability						
Economic disadvantage	1.096 ***	0.292	0.038	0.440	0.350	0.015
Percent linguistically isolated households	−0.005	0.026	−0.002	−0.023	0.027	−0.009
Percent Latina/o population				0.056 ***	0.016	0.051
Percent non-Latina/o black population				0.024	0.033	0.008
Emission sources						
Percent industrial-zoned land	0.283 ***	0.013	0.119	0.281 ***	0.013	0.118
Traffic density (1000 s)	1.451 ***	0.138	0.062	1.431 ***	0.138	0.061
Air basin regional controls						
Percent tract in South Coast	0.148 ***	0.028	0.255	0.157 ***	0.028	0.270
Percent tract in San Joaquin Valley	0.215 ***	0.029	0.216	0.212 ***	0.029	0.213
Constant	13.644 ***	2.516		12.418 ***	2.503	
Lambda (λ)	0.944 ***	0.006		0.941 ***	0.006	
Multicollinearity condition index	4.979			8.773		
Log likelihood	−30,387.997			−30,381.966		
Degrees of freedom	7603			7601		
Akaike information criterion	60,790.000			60,781.900		
Moran’s *I* of residuals ^3^	−0.006			−0.006		

^1^ Model 1 operationalizes population vulnerability solely with CalEnviroScreen 2.0 socioeconomic variables. ^2^ Model 2 adds racial composition (i.e., the percent of Latinas/os and non-Latina/o black population in a tract) to Model 1 variables. ^3^ A second-order queens adjacency spatial weights matrix was used in the regression analyses and Moran’s *I* analysis of regression residuals. Insignificance of Moran’s *I* results are based on 9999 permutations. *** *p* < 0.001 (two-tailed test).

**Table 7:** Spatial error regression results for adjusted cumulative pollution burden percentile on population vulnerability (Latina/o and black cumulative disadvantage), emission sources, and air basin regional controls (*N* = 7610 tracts).

Variables	b	S.E.	B
Population vulnerability			
Latina/o cumulative disadvantage	1.303 *	0.626	0.045
Black cumulative disadvantage	0.102	0.638	0.004
Emission sources			
Percent industrial-zoned land	0.282 ***	0.013	0.118
Traffic density (1000 s)	1.450 ***	0.138	0.061
Air basin regional controls			
Percent tract in South Coast	0.153 ***	0.028	0.264
Percent tract in San Joaquin Valley	0.213 ***	0.029	0.214
Constant	14.056 ***	2.451	
Lambda (λ)	0.942 ***	0.006	
Multicollinearity condition index	5.664		
Log likelihood	−30,384.942		
Degrees of freedom	7603		
Akaike information criterion	60,783.900		
Moran’s *I* of residuals ^1^	−0.006		

^1^ A second-order queens adjacency spatial weights matrix was used in the regression analyses and Moran’s *I* analysis of regression residuals. Insignificance of Moran’s *I* results are based on 9999 permutations. * *p* < 0.05; *** *p* < 0.001 (two-tailed test).
